# Cost-effectiveness of fondaparinux versus enoxaparin in non-ST-elevation acute coronary syndrome in Canada (OASIS-5)

**DOI:** 10.1186/s12872-015-0175-1

**Published:** 2015-12-29

**Authors:** Jorge Alfonso Ross Terres, G. Lozano-Ortega, R. Kendall, M. J. Sculpher

**Affiliations:** GlaxoSmithKline, Mississauga, ON Canada; GlaxoSmithKline, 2301 Renaissance Boulevard, King of Prussia, PA 19406 USA; Oxford Outcomes Ltd, Vancouver, Canada; Center of Helth Economics, University of York, York, UK

**Keywords:** Acute coronary syndromes, Health economics

## Abstract

**Background:**

Acute coronary syndrome (ACS) refers to a spectrum of life-threatening cardiac diseases usually due to coronary artery plaque rupture, subsequent thrombin generation plaque activation and thrombus formation. To date, no economic analyses have been published about the use of fondaparinux in NSTE-ACS patients in Canada. The purpose of our study is to estimate the lifetime cost-effectiveness of fondaparinux compared to enoxaparin for non-ST-elevation acute coronary syndrome (NSTE-ACS) patients in a Canadian hospital setting.

**Methods:**

As an extension of a previous published economic analysis for US patients, an event-based decision analytic model was constructed using clinical and resource use data from OASIS-5, a randomized trial of 20,078 patients from 41 countries. A public payer perspective in the hospital setting was adopted. Resource use data from the trial were valued using Canadian costs. A cost regression model was developed to estimate the mean cost of managing the clinical events over the 180 day period. Annual costs of long-term care for ACS patients were added after 180 days until death. Long-term survival was incorporated using Canadian life tables with further adjustment for additional risks associated with NSTE-ACS. Quality-of-life (utility) decrements from published sources were applied to clinical events. Lifetime costs (2009 CAD$) and quality-adjusted life-years (QALYs), discounted annually at 5 %, were estimated for the typical patient in OASIS-5 (i.e., at mean covariate values).

**Results:**

The trial data showed that fondaparinux is protective against all clinical events observed in the trial. The model showed that: over 180 days, fondaparinux dominates enoxaparin, producing similar estimates of QALYs gained and saving $439; over a patient’s lifetime, fondaparinux yields an ICER of $4293/QALY. Based on PSA, the probabilities that fondaparinux dominates enoxaparin (less costly and more effective) and that is cost-effective at a $50,000 threshold were 42 % and 96 %, respectively.

**Conclusions:**

In the Canadian hospital setting, fondaparinux is cost-effective when compared to enoxaparin for the treatment of NSTE-ACS. This result holds both in the immediate post-event period and over the lifetimes of patients.

**Electronic supplementary material:**

The online version of this article (doi:10.1186/s12872-015-0175-1) contains supplementary material, which is available to authorized users.

## Background

Acute coronary syndrome (ACS) refers to a spectrum of life-threatening cardiac diseases usually due to coronary artery plaque rupture, subsequent thrombin generation plaque activation and thrombus formation.

Non-ST-elevation acute coronary syndrome (NSTE-ACS) comprises 2 components: unstable angina (UA) and non-ST-elevation myocardial infarction. Pharmacological recommendations and sequence of therapy will depend on the individual management strategy and treatment guidelines, but for both invasive and conservative strategies, a combination of antiplatelet and anticoagulation agents is recommended. Most commonly, antiplatelet therapy will be comprised of aspirin plus clopidogel and/or a glycoprotein IIb/IIIa (GP) inhibitor, followed by an anticoagulant agent. Options for anticoagulation therapy include fondaparinux (Arixtra), unfractionated heparin (UFH) bivalirubin and low-molecular heparins, such as enoxaparin.

Fondaparinux is a well established synthetic anticoagulant that inhibits thrombus formation by interrupting the blood coagulation cascade through antithrombin IIIA-mediated selective inhibition of factor Xa.

The Fifth Organization to Assess Strategies in Acute Ischemic Syndromes Investigators (OASIS-5) trial randomized 20,078 patients with NSTE-ACS to fondaparinux or enoxaparin [1].

Patients were randomly assigned to a study group within 24 h after the onset of symptoms and were eligible if they met at least two of the three following criteria: an age of at least 60 years, an elevated level of troponin or creatine kinase MB isoenzyme, or electrocardiographic changes indicative of ischemia. Patients with contraindications to low-molecular-weight heparin, recent hemorrhagic stroke, indications for anticoagulation other than an acute coronary syndrome, or a serum creatinine level of at least 3 mg per deciliter (265 μmol per liter) were excluded [2]. The study protocol was approved by the respective ethics committees and regulatory bodies (ClinicalTrials.gov number, NCT00139815) Additional file 1. 

The Trial showed similar rates of ischemic events at 9 days but, by 180 days, fondaparinux reduced major bleeding and improved mortality and morbidity.

To date, no economic analyses have been published about the use of fondaparinux in NSTE-ACS patients in Canada. As an extension of a previous published analysis based on US patients [1], this article reports on an evaluation of the costs and benefits of fondaparinux and enoxaparin, in order to determine which one is more cost-effective, both in short term (180 days) and in the long term, using a life-time model in a Canadian hospital setting.

## Methods

This cost-effectiveness analysis was based on a published and well-established methodology applied to estimate the cost-effectiveness of fondaparinux versus enoxaparin in the United States hospital setting [1]. Briefly, the analysis consisted of 2 phases. First phase estimates the differential cost of fondaparinux and enoxaparin over 180 days (mean OASIS-5 follow-up). The analysis relates to a public-payer perspective in the Canadian hospital setting. The second phase assessed long-term cost-effectiveness in terms of costs and quality-adjusted life years (QALYs). Costs and QALYs are discounted at 5 % per annum.

An event-based decision analytical model was constructed using clinical and resource use data from OASIS-5 and was previously published by Sculpher et al [1]. The probabilities of death, non-fatal myocardial infarction (MI), non-fatal stroke, and major and minor bleeds over a period of 180 days, for both treatment strategies, were estimated using a set of risk equations derived from OASIS-5 data. Bleeding events are included in the cost analysis but they are assumed not to affect long-term prognosis.

In the short-term cost analysis, the differential cost of each therapy was calculated by multiplying the estimated cost of each event by the relevant event risk and summing all the products. Decrements in health-related quality of life (HRQoL) and long-term prognoses estimates were used for the long-term cost-effectiveness analysis. A 5 % annual discount rate was applied to all costs and QALYs.

Clinical events and resource use over a mean follow-up of 173 days (range, 90–180 days) where used from the OASIS-5 trial. This trial involved 20,078 patients with NSTE-ACS which were randomized to either fondaparinux (2.5 mg daily) or enoxaparin (1 mg per kg twice daily) for a mean of 5 days. This was a global study in which 41 countries participated including multiples hospitals Canada. The primary end point of death, MI or refractory ischemia at 9 days, was similar between treatments, but major bleeding at 9 days was lower with fondaparinux [1]. The trial found significantly lower rates of death and nonfatal events with fondaparinux at 180 days [1].

The differential cost of fondaparinux and enoxaparin was based on the acquisition costs of the 2 therapies and the product of 2 sets of estimates from the trial (i) risks of key clinical events: death, nonfatal MI, nonfatal stroke, a combination of MI and stroke and major and minor bleeds (ii) the mean costs of these events. The 180-day probabilities of clinical events used OASIS-5 data and were based on parametric survival modeling using a Weibull [3] distribution. The hazard of each event is estimated as a function of treatment and baseline covariates. The choice of covariates has been based on the clinical judgment of and variables used in the TIMI [4] and GRACE [4] risk scoring systems.

Key resource use data in OASIS-5 included study drugs, concomitant medications, and inpatient days. The base-case analysis uses resource use data from the 1403 Canadian patients valued using Canadian costs (2009 $CAD), largely based on data obtained through the Ontario Health Insurance Program (OHIP) [5–7]. These costs include: medications, laboratory and diagnostic procedures, therapeutic services, and primary and secondary diagnoses, total costs (fixed and variable) to the hospital. Daily room costs are included for inpatient interventions. In the case of procedures and interventions, fees to medical professionals are estimated using the Ontario Case Costing Initiative (OCCI), OHIP and the published literature [8–10]. Drug costs are derived from the Ontario Drug Benefit (ODB) formulary. Blood transfusion costs are from ODB formulary and published sources [11].

In order to estimate the mean cost over 180 days of: (i) patients without clinical events, and (ii) the additional cost associated with each event, regression modeling was used. An ordinary least squares model was used with a series of dummy variables used to represent events. Concomitant drug costs were based on the mean dosage in OASIS-5 and the mean therapy duration in Canadian trial patients. In the base case, these costs are based on the ODB formulary.

Patients remaining alive at 180 days will survive, though with some decrements to their health, for a time depending on sex, age and health state at 180 days. Patients will also continue incurring costs for the treatment of their heart disease. Long-term cost-effectiveness assumes that the clinical differences between the alternative antithrombotics ceases at 180 days.

Since the OASIS-5 trial did not collect any data on HRQoL, estimates are derived from other published sources and applied to clinical events. Due to the unavailability of Canadian-specific values, estimates were derived from age-and sex-specific “population norms” for the United States (US) based on EQ-5D instrument [12].

Long-term mortality rates are incorporated using Canadian life tables. The additional mortality risk in NSTE-ACS is quantified in terms of a relative risk compared with the general population and distinguishes between patients with a nonfatal MI, a nonfatal stroke of any severity, both types of nonfatal event or neither within 6 months of their ACS episode. This uses data from the United Kingdom UK PRAIS study [13]. Beyond the initial 180-day period, the cost of long-term care for ACS patients was assumed to be $10,783 per annum [14].

Mean (expected) cost-effectiveness of the probability of each therapy being the least costly, and the more cost-effective therapy assuming a cost-effectiveness threshold of $50,000 per QALY gained is presented using probabilistic sensitivity analysis (PSA) [15]. Short-term costs and long-term cost-effectiveness are shown for the “average” trial patient. Also, results are calculated for national patients at high and low risk of the composite event of death and nonfatal MI and stroke with enoxaparin over 180 days, assuming the relative effect of fondaparinux remains unchanged.

Lifetime costs and (QALYs) were estimated for the typical patient in OASIS-5 (i.e., at mean covariate values), as well as for a patient at the 2.5th and 97.5th percentiles of composite risk of death, nonfatal MI or nonfatal stroke. A cost analysis was performed at 180 days, and incremental cost-effectiveness ratios (ICERs) were output over the entire lifetime. PSA was carried out to assess parameter uncertainty.

## Results

The risk equations showed consistent results with the clinical analysis [2]. For each type of event over the 180-day follow-up period, fondaparinux was protective compared with enoxaparin, although the effect was not statistically significant for nonfatal MI. (See Table 1 in [1]). Table 1 shows key resource use over the 180-day follow up in OASIS-5 in Canadian patients, together with estimated unit costs. Resource use was very similar between enoxaparin and fondaparinux patients; the main difference between fondaparinux and enoxaparin was in the rate of percutaneous coronary intervention. Also, a higher proportion of Canadian subjects needed blood transfusion under fondaparinux, although this difference was not statistically significant (relative risk (RR) of having a blood transfusion: 1.21; 95 % confidence interval: 0.80, 1.82). When considering all patients randomized in the trial, the RR of having a blood transfusion was 0.75 (0.65 to 0.86).

**Table 1:** Summary of key resource use observed in the OASIS-5 trial patients randomized in Canada (*n* = 1.403) within the 180-day follow-up period together with associated unitcosts (adapted from Table 2 in US analysis’)

	Fondaparinux	Enoxaparin	
Item of resource use	(*n* = 701)	(*n* = 7 02)	Unit cost (CAD 2009 S)
Sudy drugs (mean (SD) days)	4.10 (2.28)	3.65 (2.11)	Enoxaparin: 15 per day ^a^
			Fondaparinux: 15 per day ^b^
Days in hospital (mean (SD))			
ICU	3.54 (5.07)	3.57 (3.83)	1433 per day
General ward	4.64 (10.13)	4.46 (9.61)	547 per day
Step down	2.98 (5.01)	2.85 (5.28)	547 per day
Selected concomitant medicines			
Clopidcgrel (mean (SD) days)	105.25 (77.18)	106.07 (77.69)	2.58 per day
Ticlopidine (mean (SD) days)	0.08 (1.75)	1.04 (12.14)	0.55 per day
Glycoprotein llb/llla antagonists (n (%))	201 (28.67)	212 (30.20)	1094
Selected procedures (n (%) with one or more)	47 (6.70)	39 (5.56)	543 per procedure
Blood transfusion	603 (86.02)	607 (86.47)	5401 per procedure
Coronary angiography	298 (42.51)	335 (47.72)	10,543 per procedure
Percutaneous coronary intervention	143 (20.40)	134 (19.09)	21,286 per procedure
Coronary artery bypass graft			

*SD* Standard deviation, *n* Number, *ICU* Intensive care unit

^a^Based on 80 mg injection

^b^Based on 2.5 mg injection

Table 2 presents the results of the short-term cost analysis. The total expected costs of the two therapies over 180 days shows fondaparinux was associated with potential cost savings, although this finding was not statistically significant.

**Table 2:** Six-month cost analysis comparison of the expected costs of enoxaparin and fondaparinux over six months (estimates relate to a patient with “average” characteristics [4], adapted from Table 2 in US analysis’)

	Cost of event^b^ (95 % Cl)	Probability of event (95 % Cl)		Cost per patient^c^ (95 % Cl) (2009 CADS)	
Event/resource use	(2009 CAD $)	Enoxaparin	Fondaparinux	Enoxaparin	Fondaparinux
Death	−691 (−4943, 3487)	0.046 (0.042, 0.050)	0.041 (0.038, 0.045)	−28 (−225, 157)	−2 5 (−203, 141)
Non-fata Ml	15,021 (10,997, 19,053)	0.049 (0.046, 0.055)	0.047 (0.043, 0.052)	752 (527, 978)	712 (505, 927)
Non-fatal stroke	18,755 (10,698, 26,864)	0.011 (0.009, 0.014)	0.007 (0.006, 0.009)	217 (113, 340)	141 (76, 226)
Non-fatal Ml & Stroke	3407 (−29,232, 35,980)	0.0006 (0.0005, 0.0007)	0.0003 (0.0003, 0.0005)	2 (−17, 20)	1 (−10, 13)
Major bleed	17,553 (13,827, 21,023)	0.052 (0.048, 0.057)	0.038 (0.034, 0.042)	921 (728, 1126)	665 (524, 823)
Minor bleed	3604 (171, 7182)	0.071 (0.066, 0.077)	0.036 (0.032, 0.040)	257 (10, 521)	129 (5, 254)
Enoxaparin Treatment	60	1	0	60	0
Fondaparinux Treatment	63	0	1	0	63
Other costs^d^	24,143 (22,785, 25,425)	1	1	24,143 (22,785, 25,425)	24,143 (22,785, 25,425)
Tota mean cost				26,302 (25,042, 27,473)	25,864 (24,689, 27,025)
*Difference in mean costs*					*$-439 (−2069, 1322)*

*Ml* Myocardial infarction, *Cl* Confidence interval, *CAD* Canadian dollars

^a^Covariates at mean values are Age 67.1; proportion male 0 62; proportion with history’ of: heart failure 0 14. diabetes 0 25. hypertension 0 67. ST depression 0 51; creatinine quartiles quartile 1 0 26. quartile 2 0 26. quartile 3 0 25. quartile 4 0 23

^b^Results of the cost regression relating to patients randomized in Canada (*n* = 1.403) (2009 CAD $)

^c^Estimated by multiplying the probability of event times cost of event, when performing the probabilistic sensitivity analysis

^d^Background cost associated with patients who experienced ischemic heart disease

The long-term cost-effectiveness analysis reported in Table 3 showed that the ICER is well below conventional thresholds for the 3 types of patients considered in the model (patients of average characteristics, and patients at low and high risk of the composite event of death, MI or stroke, respectively). The uncertainty in these results is shown in terms of the probabilities that fondaparinux is cost saving, and cost-effective at a threshold of $50,000 per QALY gained; these probabilities ranged from 34 to 48 % and 83 to 97 %, respectively.

**Table 3:** Cost-effectiveness results over a lifetime time horizon (adapted from Table 2 in US analysis)

	Enoxaparin	Fondaparinux	ICER
Patient with average characteristics			
Expected cost	$110,477	$110,661	$4293
Expected quality-adjusted life years	6.37	6.42	
Probablity most cost-effective at a threshold of $50,000 per QALY		96 %	
Probability of cost-saving		42 %	
Patient at low risk of composite event over 180 days (2.5^th^ percentile)			
Expected cost	$164,824	$164,836	$661
Expected quality-adjusted life years	11.01	11.03	
Probability most cost-effective at a threshold of $50,000 per QALY		83 %	
Probability of cost-saving		48 %	
Patient at high risk of composite event over 180 days (97.5^th^ percentile)			
Expected cost	$70,846	$71,299	$4666
Expected quality-adjusted life years	3.37	3.47	
Probability most cost-effective at a threshold of $50,000 per QALY		100 %	
Probability of cost-saving		34 %	

## Discussion

After assessing the cost-effectiveness of the alternative therapies reflecting Canadian practice based on resource use, unit costs, age- and sex-specific population, mortality risks and HRQoL data specific to the country, we found that fondaparinux is cost-effective and, under most scenarios, a dominant strategy compared with enoxaparin. In part, this reflects not only the lower acquisition cost of fondaparinux but also the lower rates of clinical events during the 6-month period after treatment.

There are limitations to modeling the cost-effectiveness of products studied in multinational trials because studies are powered on overall event rates, not event rates in individual countries, yet local country resource use and cost data are required for decision making. Therefore, to inform payers in Canada, the model was based on Canadian resource use from the trial, while using whole trial event rates.

At baseline in OASIS-5, Canadian patients were generally similar to those in the overall trial with some differences with the US population which were less likely to have unstable angina (rather than MI), had undergone more revascularization procedures, were more likely to be diabetic, and were less likely to have ST-segment depression. Differences in medical care between Canada and the United States have been reported in the contemporary management of patients with non– ST-elevation MI and UA. Several studies have reported findings in which angiography, angioplasty, and bypass surgery are more common in the United States than in Canada. The differences could be explained in the availability on-site facilities in different institutions across Canada which could lead to longer waiting times which could have an impact on long outcome of this population. This could be reflection of longer stay in ICU and general ward [16].

As previously published in the original analysis performed in the US population, fondaparinux resulted in cost savings to the hospital [1]. When 180-day cost and clinical results were extrapolated to long-term cost-effectiveness, fondaparinux was dominant (less costly and more effective in terms of QALYs under most scenarios.

The OASIS-5 trial reported that in the subgroup of patients undergoing percutaneous coronary intervention (PCI), fondaparinux reduced major bleeding by one half while maintaining similar efficacy to enoxaparin. This resulted in superior net clinical benefit of fondaparinux relative to enoxaparin in PCI patients. In this analysis, both enoxaparin and fondaparinux were associated with catheter thrombosis in 1 % of patients. Consequently, it is recommended that, in patients treated with fondaparinux for ACS, adjunctive UFH (50–60 IU/kg bolus) be used in place of an IV fondaparinux bolus just before the PCI procedure. It has been shown in OASIS-5 that, even with the use of supplemental UFH in PCI patients, the reduction in bleeding with fondaparinux appears to be preserved [1].

Given this, and the modest cost of UFH relative to IV fondaparinux, the use of adjunctive UFH can be expected to have little effect on the cost-effectiveness results presented here.

## Conclusions

This analysis showed that differences in clinical events over 180 days have long-term prognostic implications. In the Canadian hospital setting, fondaparinux is cost-effective when compared to enoxaparin for the treatment of NSTE-ACS. This result holds both in the immediate post-event period and over the lifetimes of patients.

## Additional file

Additional file 1:
**Affiliation of Ethics commitees.** (DOCX 123 kb)

## Abbreviations

ACS: acute coronary syndrome
CAD$: Canadian dollars
GP: glycoprotein IIb/IIIa
HRQoL: health-related quality of life
ICERs: incremental cost-effectiveness ratios
MI: myocardial infarction
NSTE-ACS: non-ST-elevation acute coronary syndrome
OASIS-5: Organization to Assess Strategies in Acute Ischemic Syndromes Investigators
OCCI: Ontario Case Costing Initiative
ODB: Ontario Drug Benefit
OHIP: Ontario Health Insurance Program
PSA: probabilistic sensitivity analysis
QALYs: Quality-adjusted life-years
RR: Relative risk
UA: unstable angina
UFH: unfractionated heparin
US: United States
