# Supplementary material for: Cost-effectiveness of fondaparinux versus enoxaparin in non-ST-elevation acute coronary syndrome in Canada (OASIS-5)
Source: BMC Cardiovasc Disord. 2015 Dec 29;15:180. doi: 10.1186/s12872-015-0175-1 (PMC4696110; doi:10.1186/s12872-015-0175-1)
Supplement: Additional file 1: — Affiliation of Ethics commitees. (DOCX 123 kb) [file 12872_2015_175_MOESM1_ESM.docx]

**Affiliations of all the ethics committees that approved the study**

| **ARGENTINA** | **IRB** |
| --- | --- |
|  | Comité de Docencia e Investigación de la  Corporacion |
|  | Comité de Docencia e Investigación  Clínica Coronel Suárez |
|  | Comité de Docencia e Investigación de la Clinica  Privada Milenio |
|  | Comité de Etica de la Policlínica Bancaria |
|  | Comité de Docencia e Investigación del Htal Luis C. Lagomaggiore y Comité de Etica en Investigacion del Htal.Luis C.lagomaggiore |
|  | Comité de Docencia e investigación |
|  | Comité de Docencia e Investigación -Sanatorio  Médico de Diagnóstico y Tratamiento |
|  | Comité de Docencia e Investigación del Centro  Privado de Cardiología de Tucumán |
|  | Comité de Docencia e Investigación del Hospital  Centro de Salud |
|  | Comité de Docencia e Investigación del ICR |
|  | Comité de Bioética y Comite de Docencia e  Investigación del Hospital General de Agudos |
|  | Comité de Docencia e Investigación del Santorio  Antártida |
|  | Comité de Docencia e Investigación del Sanatorio  Modelo de Quilmes |
|  | Comité Independiente de Etica para Ensayos en  Farmacología Clínica |
|  | Comité de Docencia e Investigación de la Clínica |
|  | Comité de Docencia e Investigación y el Comité de  Bioética del Hospital Italiano de la Plata |
|  | Comisión de Investigación del Hospital San Bernardo de Salta |
|  | Comité de Docencia e Investigación del Instituto  Cardiovascular de San Luis Rivadavia |
|  | Comité de Revisión Interna dependiente del  Departamento de Docencia e Investigación |
|  | Comité Institucional de Etica de Investigación en  Salud del Sanatorio Allende Hipólito Yrigoyen |
|  | Comité Institucional de Etica de la Investigación en |
|  | Comité de Etica Medica y Comité de Docencia e  Investigación del Hospital Francés |
|  | Comité de Docencia e Investigación del Hospital  Británico |
|  | Comité de Etica en Investigación del CEMIC |
| **AUSTRALIA** | **IRB** |
|  | Flinders Clinical Research Ethics Committee and  Clinical Drug Trials Committee |
|  | Ethics of Human Research Committee  The Queen Elizabeth Hospital and Health Service |
|  | Concord Hospital Ethics Committee |
|  | Sydney West Area Health Service Ethics Committee |
|  | Hunter Area Research Ethics Committee  John Hunter Hospital |
|  | Human Research Ethics Committee  Royal North Shore Hospital |
|  | Mid North Coast Area Health Service Research  Ethics Committee |
|  | Human Ethics Research Committee  Illawarra Area Health Service  University of Wollongong |
|  | Human Research Ethics Committee  The Prince Charles Hospital Health Service District |
|  | Uniting HealthCare Human research Ethics  Committee |
|  | Human Research Ethics Committee  Royal Brisbane and Womens Hospital |
|  | Human Research Ethics Committee  Royal Brisbane and Womens Hospital |
|  | Research Ethics Committee Princess Alexandra Hospital |
|  | Cairns Base Hospital Ethics Committee  Cairns Base Hospital |
|  | Research and Ethics Committee Princess Alexandra Hospital |
|  | Human Research Ethics Committee  The Rockhampton Health Service District  Rockhampton Hospital |
|  | Townsville Health Service District Institutional Ethics  Committee |
|  | Gold Coast Hospital Ethics Committee  Queensland Health, Gold coast Hospital |
|  | Research and Ethics Advisory Committee  The Geelong Hospital |
|  | Eastern Health Research and Ethics Committee |
|  | Austin Health Human Research Ethics Committee  Research Support Unit  North Wing, Heidelberg Repatriation Campus  Heidelberg Repatriation Hospital |
|  | Central Gippsland Health Service Ethics Committee  Central Gippsland Health Service |
|  | Human Research Ethics Committee  Locked Bag 29 |
|  | Human Research Ethics Committee of Bendigo  Bendigo Health Care Group |
|  | Human Research Ethics Committee  Royal Melbourne Hospital |
|  | Human Research Ethics Committee  Royal Perth Hospital |
|  | Human Research Ethics Committee Sir Charles Gairdner Hospital |
|  | Joondalup Health Campus Ethica Committee |
|  | Fremantle Hospital  Health Service Human Research Ethics Committee |
| **AUSTRIA** | **IRB** |
|  | Ethikkommission der Stadt Wien |
| **BELGIUM** | **IRB** |
|  | AZ Middelheim  Commissie voor Medische Ethiek  ZNA/O.C.M.W. Antwerpen |
|  | Centre Hospitalier Régional de Huy - Comité d'éthique médicale |
|  | C.E. Biomédicale Hospito-Facultaire U.C.L. Tour Harvey Avenue Hippocrate |
|  | Ethics Committee  AZ Sint-Jan AV, rudderhove 10 |
|  | Ethics Committee  Imeldaziekenhuis VZW |
|  | Comité d'éthique des centres Hospitaliers Jolimont  Lobbes et Nivelles  Tubize OM 20, Siège Jolimont |
|  | Ethische Commissie Ziekenhuis Oost-Limburg |
| **BRAZIL** | **IRB** |
|  | Comitê de Ética em Pesquisa da Faculdade de  Medicina  Universidade Federal da Bahia |
|  | Comitê de Ética em Pesquisa da Universidade de  Santo Amaro - UNISA  Rua Prof. Enéas de Siqueira |
|  | Comitê de Ética em Pesquisa do Hospital  Universiatario Perdro Ernesto |
|  | Comitê de Ética em Pesquisa da Santa Casa de |
|  | Comitê de Ética em Pesquisa do Instituto de  Moléstias Cardiovasculares |
|  | Comitê de Ética em Pesquisa da Santa Casa de  Pelotas |
|  | Comitê de Ética em Pesquisa da Sociedade  Evangélica Beneficente de Curitiba |
|  | Comitê de Ética em Pesquisa da Faculdade de Medicina de São José do Rio Preto |
|  | Comitê de Ética em Pesquisa do Instituto de  Cardiologia de Santa Catarina Rua Adolfo Donato da Silva |
|  | Comitê de Ética em Pesquisa da UNIFESP/HSP Rua Botucatu |
|  | Comitê de Ética em Pesquisa do Hospital Pró  Cardíaco |
|  | Comitê de Ética em Pesquisa com Seres Humanos para a área da Saúde da PUC |
|  | Comitê de Ética em Pesquisa do Centro de Ciências  Médicas e Biológicas da PUC/SP |
|  | Comitê de Ética em Pesquisa do Instituto Dante  Pazzanese de Cardiologia |
|  | Comitê de Ética em Pesquisa da Irmandade Santa  Casa de Misericórdia de Porto Alegre |
|  | Comitê de Ética em Pesquisa Clínica do Instituto  Nacional de Cardiologia Laranjeiras  Rua das Laranjeiras |
|  | Comitê de Ética em Pesquisa do Hospital São Lucas da PUCRS |
|  | Comitê de Ética em Pesquisa do Hospital das  Clínicas de Ribeirão Preto da Universidade de São |
|  | Hospital das Clínicas  Rua Dr. Ovídio Pires de Campos |
|  | Comitê de Ética da Fundação Universidade Estadual de Maringá Fundação Universidade Estadual de Maringá - Pró-Reitoria de Pesquisa e Pós-  Graduação |
|  | Comitê de Ética em Pesquisa Clínica da Santa Casa de Misericórdia de Belo Horizonte |
|  | Faculdade de Ciências Médicas  Comitê de Ética em Pesquisas - Universidade |
|  | Comitê de Ética em Pesquisa envolvendo Seres Humanos da Faculdade de Medicina de Marília |
|  | Comitê de Ética em Pesquisa do Instituto de  Cardiologia do Rio Grande do Sul/ Fundação  Universiitária de Cardiologia |
|  | Comitê de Ética em Pesquisa do Hospital Socor |
| **CANADA** | **IRB** |
|  | Capital Health Research Ethics Board |
|  | Lakeridge Health Corporation Research Ethics Board |
|  | The Scarborough Hospital |
|  | Comite D'ethique de la Recherche de L'hopital  Maisonneuve |
|  | The Scarborough Hospital |
|  | Clinical Research Ethics Board  The University of British Columbia |
|  | Southlake Regional Health Center Clinical Trials  Committee |
|  | Research Committee of Rouge Valley Health Care  System |
|  | Comite d'Ethique de la Recherche du Centre  Hospitalier Pierre-Le Gardeur |
|  | Comite d'Ethique de la Recherche |
|  | William Osler Health Centre REC |
|  | Biomedical D Research Ethics Board |
|  | South Fraser Regional Research Review Committee |
|  | Comite d'ethique a la recherche |
|  | Research Ethics board, Sunnybrook Campus |
|  | Conjoint Health Research Ethics Board |
|  | Hamilton Health Sciences Henderson Campus |
|  | Comite D'Ethique et de la Recherche |
|  | Lakeshore General Hospital |
|  | Tri-Hospital Research Ethics Board |
|  | Biomedical Research Ethics Board |
|  | Institutional Review Board |
|  | Comite d'Ethique de la Recherche CHUM |
|  | Hamilton Health Sciences/McMaster University REB |
|  | South Fraser Regional Research Review Committee |
|  | Comite d'Ethique de la Recherche |
|  | Biomedical Research Ethics Board  University of Saskatchewan |
|  | Centre de sante et de services sociaux de la region de Thetford |
|  | Biomedical D Research Ethics Board |
|  | Comite d'Ethique de la Recherche du CHUM |
|  | Hamilton Health Sciences/McMaster Health Sciences  REB |
|  | Conjoint Health Research Ethics Board  University of Calgary |
|  | St. Catherines General Site Research Ethics Board |
|  | Fraser Health Authority  Fraser North Area Royal Columbian Hospital |
|  | Research Review & Ethical Approval Committee  Memorial Pavilion, Kenning Wing |
|  | Ottawa Hospital Research Ethics Board |
|  | UBC/Providence Health Care Research Ethics  Board |
|  | Bioethics Committee Niagara Health System Greater Niagara General Site |
|  | Health Research Ethics Board  University of Alberta |
|  | Office of Research Ethics  The University of Western Ontario |
|  | Thunder Bay Regional Health Sciences Centre |
|  | Conjoint Health Research Ethics Board Rm 93  Heritage medical Research Bldg. |
|  | Human Investigation Committee |
|  | Institutional Research Review Committee |
|  | Comite de'Ethique de la Recherche Clinique du  CHUL |
|  | Comite d'ethique de la recherche du CHUM |
|  | Tri-Hospital Research Ethics Board  Grand River Hospital |
|  | Reseau Sante Richelieu-Yamaska |
|  | UOHI Human Research Ethics Board |
|  | Comite d'ethique de la recherche Hopital du Sacre- Coeur de Montreal |
|  | Office of Research Ethics  The University of Western Ontario |
|  | Comite d'ethique de la Recherche  Hopital de l'Enfant-Jesus des CHA |
|  | Research Ethics Committee |
|  | Centre de Recherche Clinique du CHUS Hopital Fleurimont |
| **CHILE** | **IRB** |
|  | Comite Independentie de Etica para Ensayos en  Farmacologia Clinica |
| **CHINA** | **IRB** |
|  | IEC of Zhongshan Hospital, Fudan University |
| **CROATI** | **IRB** |
|  | **Etičko povjerenstvo**  **Klinički bolnički centar Rijeka** |
|  | **Etičko povjerenstvo Klinička bolnica Split Spinčićeva 1** |
| **CZECH REPUBLIC** | **IRB** |
|  | Etická komise FN Motol |
|  | Multicentrická etická komise |
|  | Etická komise Nemocnice Slaný |
|  | Etická komise Nemocnice Podlesí |
|  | Etická komise Nemocnice Třebíč |
|  | Regionální etická komise  Okresní nemocnice Tábor |
|  | Etická komise FN Královské Vinohrady |
|  | Etická komise FN a LF UK v Plzni |
|  | Etická komise Nemocnice České Budějovice  . |
|  | Etická komise VFN a 1. LF UK v Praze  . |
|  | Lokální Etická komise při Sdružení smluvních lékařů |
|  | Multicentrická etická komise  Krajská nemocnice Liberec |
| **DENMARK** | **IRB** |
|  | Den Videnskabsetiske Komité for Århus Amt |
| **ESTONIA** | **IRB** |
|  | Ethics Review Committee (ERC) ON Human  Research of the University of Tartu  Lembit Allikmets |
| **FINLAND** | **IRB** |
|  | The Ethics Committee of the Health Care District of  Helsinki and Uusimaa (HUS) |
| **FRANCE** | **IRB** |
|  | CCPPRB Franche-Comté service St.Joseph Hôpital  Saint Jacques |
| **GERMANY** | **IRB** |
|  | Ethikkommission der Charite Berlin |
|  | Ethik-Kommission bei der Landesärztekammer  Hessen |
|  | Ethikkommission der Medizinischen Hochschule  Hannover |
|  | Ethik-Kommission der Martin-Luther-Universität Halle- Wittenberg |
|  | Ärztekammer des Saarlandes  Ethikkommission |
|  | Bayerische Landesärztekammer  Ethikkommission |
|  | Ethikommission der Ärztekammer Nordrhein |
|  | Universität Witten / Herdecke  Ethik-Kommission |
|  | Ethik-Kommission an der Medizinischen Fakultät der  RWTH Aachen |
|  | Ethik-Kommission bei der Landesärztekammer  Hessen |
|  | Ruprecht-Karls-Universität  Medizinische Fakultät Heidelberg  Ethikkommission |
|  | Ethikkommission der Medizinischen Fakultät der Ludwigs-Maximilians Universität |
|  | Ethik-Kommission bei der Landesärztekammer  Hessen |
|  | Ethik-Kommission der Ärztekammer Hamburg |
|  | Landesärztekammer Rheinlnad-Pfalz  Ethikkommission |
|  | Landesärztekammer Baden-Württemberg  Ethikkommission |
|  | Ethikkommission der Charite Berlin |
|  | Landesärztekammer Rheinlnad-Pfalz  Ethikkommission |
|  | Ethikommission der Ärztekammer Nordrhein |
|  | Ethikommission der Ärztekammer Westfalen-Lippe  Von-Esmarch |
|  | Ethik-Kommission der Ärztekammer Niedersachsen |
| **GREECE** | **IRB** |
|  | Ethics Committee of the General Peripheral Hospital of Athens "G. Gennimatas" |
|  | Ethics Committee of the General District Hospital of  Rhodes |
|  | Ethics Committee of the General Hospital of Kavala |
|  | Ethics Committee of the 'General Peripheral Hospital of Pireaus "Tzanio" |
|  | Ethics Committee of the General Peripheral  University Hospital of Patras |
|  | Ethics Committee of the 'General Peripheral Hospital of Nikaia Pireaus "Ag. Panteleimon" |
|  | Ethics Committee of theGeneral Peripheral Hospital of Patras "Ag. Andreas" |
| **HONG KONG** | **IRB** |
|  | Institutional Review Board of the University of Hong  Kong |
|  | Institutional Review Board/ Hospital Authority Hong  Kong East Cluster |
|  | Joint the Chinese University of Hong Kong – New Territories East Cluster Clinical Research Ethics Committee (Joint CUHK-NTEC CREC) |
|  | Clinical Research Ethics Committee of the Kowloon  West Cluster (KWC-CREC) |
| **HUNGARY** | **IRB** |
|  | Gotsegen György Országos Kardiológiai  IntézetIntézeti Kutatás Etikai Bizottság |
|  | Károlyi Sándor Kórház, Etikai Bizottság |
|  |  |
|  | Fövárosi Szent István Kórház |
|  | Szent Imre Kórház Klinikai Farmakológiai Regionális  Etikai Bizottsága |
|  | Budai Irgalmasrendi Kórház Intézményi Kutatásetikai  Bizottság  Gyeney |
|  | Semmelweis Egyetem Budapest Regionális  Tudományos és Kutatásetikai Bizottság |
|  | Zala Megyei Kórház Intézményi Kutatásetikai  Bizottsága |
|  | Vas Megyei Önkormányzat, Markusovszky Kórház  Regionális Kutatásetikai Bizottság |
|  | Intézeti Etikai Bizottság Karolina Kórház- Rendelőintézet |
|  | Komárom-Esztergom Megyei Önkormányzat Szent  Borbála KórházaIKEB/IRB |
|  | Fejér Megyei Szent György KórházKutatásetikai  Bizottság |
|  | Réthy Pál Kórház-Rendelőintézet Intézeti  Kutatásetikai Bizottsága |
|  | Pándy Kálmán Megyei Kórház, GyulaIntézményi  Humán Kutatásetikai Bizottság |
|  | Jász-Nagykun-Szolnok Megyei  Hetényi Géza Kórház Rendelőintézet Etikai BizottságSzolnok |
|  | Bács-Kiskun Megyei Önkormányzat Kórháza  Intézményi Kutatás Etikai Bizottsága |
|  | Tolna Megyei Kórház |
|  | Orvostudományi Központ Regionális Kutatásetikai  Bizottság |
|  | BAZ Megyei Kórház I. Kutatásetikai Bizottság |
|  | DEOEC Regionális és Intézményi Kutatásetikai  Bizottság |
|  | Szent János Kórház Etika Bizottság |
|  |  |
| **INDIA** | **IRB** |
|  | Independent Ethics Committee |
|  | Ethics Committee  Christian Medical College |
|  | Ethics Committee  King George Medical University  Lucknow |
|  | Ethics Committee  Batra Hospital Research Institute |
|  | Ethics Committee S.P.Medical College |
|  | Ethics Committee  MGM Medical College & M Y Hospital  Division of Cardiology |
|  | Ethics Committee  Avanti Institute of Cardiology |
|  | Ethics Committee  S.A.L Hospital and Medical Institute  Opp. Doordarshan Tower |
|  | Ethics Committee  K.E.M. Hospital  Acharya Donde Marg |
|  | Ethics Committee  Medciti Hospital |
|  | Ethics Committee  St. John’s Medical College Hospital |
|  | Ethics Committee  Rajamuthaiah Medical College & Hospital  Annamalai University |
|  | Ethics Committee  Sri Ramachandra Hospital  Cardiac Care Centre |
|  | Ethics Committee  Amrita Institute of Medical Sciences and Research  Centre |
|  | Baby Memorial Hospital |
| **ITALY** | **IRB** |
|  | COMITATO ETICO INDIPENDENTE DELL'AZIENDA CASTIGLIONE 29 |
|  | COMITATO DI BIOETICA DEL POLICLINICO SAN MATTEO DI PAVIA-VIALE |
|  | COMITATO ETICO DELLA AZIENDA OSPEDALIERA DELLA PROVINCIA DI LODI |
|  | COMITATO ETICO PER LA SPERIMENTAZIONE CLINICA DEI FARMACI DELL'AZIENDA USL 2 DI  LUCCA |
|  | COMITATO ETICO PER SPERIMENTAZIONI DEI FARMACI (CESF) DELL'AZIENDA USL 8 DI AREZZO -CURTATONE |
|  | SOTTOCOMITATO ETICO PER LA SPERIMENTAZIONE CLINICA DEI FARMACI DELL'ASL 6 DI LIVORNO |
|  | COMITATO ETICO DEL POLICLINICO S.ORSOLA - MALPIGHI DI BOLOGNA |
|  | COMITATO ETICO DELL'AZIENDA ULSS 13 DI MIRANO |
|  | COMITATO ETICO PER LA SPERIMENTAZIONE CLINICA DEI FARMACI DELL'AZIENDA ULSS 21 DI  LEGNAGO |
|  | COMITATO DI BIOETICA DELL'USL 1 DI SASSARI - SARDEGNA |
|  | COMITATO ETICO DELL'ULSS 20 DI VERONA - |
|  | COMITATO INDIPENDENTE DI ETICA DELL'AZIENDA OSPEDALIERA 'CARLO POMA' DI  MANTOVA |
|  | COMITATO ETICO DELL'AZIENDA OSPEDALIERA  'G. BROTZU' DI CAGLIARI |
|  | COMITATO ETICO DEL CENTRO CARDIOLOGICO DI MILANO |
|  | Comitato Etico dell'ASL ROMA G |
|  | COMITATO ETICO DELL'UNIVERSITA' CATTOLICA DEL S. CUORE - POLICLINICO GEMELLI |
|  | COMITATO ETICO DELL'ASL DI RIMINI - EMILIA ROMAGNA - VIA SETTEMBRINI 2 -47900 - RIMINI MARIO MARZALONI |
|  | COMITATO ETICO DELLE AZIENDE SANITARIE DELL'UMBRIA - UMBRIA |
|  | Comitato Etico dell'Azienda USL ROMA H di Albano  Laziale |
|  | COMITATO ETICO DELL'AZIENDA SANITARIA N.13 DI ASCOLI PICENO |
|  | COMITATO ETICO DELL'OSPEDALE CASA SOLLIEVO DELLA SOFFERENZA (IRCCS) DI SAN  GIOVANNI ROTONDO |
|  | COMITATO ETICO PROVINCIALE DI MODENA - VIA DEL POZZO 71 |
|  | COMITATO ETICO DELL'ASL LE/1 DI LECCE |
|  | COMITATO ETICO DELL'AZIENDA OSPEDALIERA - VIA CANDIANI |
|  | COMITATO ETICO INDIPENDENTE DELL'AZIENDA OSPEDALIERA 'S. MARIA DELLA MISERICORDIA' DI |
|  | COMITATO ETICO INDIPENDENTE (IEC) DELL`AZIENDA SANITARIA LOCALE DELLA PROVINCIA DI MILANO |
|  | COMITATO DI BIOETICA DELL'AZIENDA OSPEDALIERA ''V. CERVELLO'' DI PALERMO |
|  | COMITATO ETICO DELL'ASL DI RAVENNA |
| **NORWAY** | **IRB** |
|  | Regional komité for medisinsk forsknigsetikk, Helseregion Øst |
| **POLAND** | **IRB** |
|  | Komisja Bioetyczna przy CMKP , Szpital im. Orlowskiego |
|  | Komisja Bioetyczna przy Akademii Medycznej w  Warszawie |
|  | Komisja Bioetyczna przy Okregowej Izbie Lekarskiej w Warszawie, |
|  | Komisja Bioetyczna przy Okregowej Izbie Lekarskiej w Lodzi, |
|  | Komisja Bioetyczna przy Okregowej Izbie Lekarskiej w Gdansku, |
|  | Komisja Bioetyczna przy Slaskiej Izbie Lekarskiej w  Katowicach |
|  | Komisja Bioetyczna przy Okregowej Izbie Lekarskiej w Opolu |
|  | Komisja Bioetyczna przy Dolnoslaskiej Izbie  Lekarskiej we Wroclawiu |
|  | Komisja Biotyczna przy Okregowej Radzie Lekarskiej w Krakowie |
|  | Komisja Bioetyczna przy Okregowej Izbie Lekarskiej w Tarnowie, |
|  | Komisja Bioetyczna Okregowej Rady Lekarskiej w  Lublinie |
|  | Komisja Bioetyczna przy Swietokrzyskiej Izbie  Lekarskiej w Kielcach |
|  | Komisja Bioetyczna przy Okregowej Izbie Lekarskiej w Czestochowie, |
|  | Komisja Bioetyczna przy Kujawsko-Pomorskiej Izbie  Lekarskiej w Toruniu |
|  | Komisja Bioetyczna przy Bydgoskiej Izbie Lekarskiej w Bydgoszczy |
|  | Komisja Bioetyczna przy Akademii Medycznej im. K. Marcinkowskiego w Poznaniu, |
| **PORTUGAL** | **IRB** |
|  | Comissão de Ética do Hospital Infante D. Pedro |
|  | Comissão de Ética do Hospital Fernando Fonseca |
|  | Comissão de Ética do Hospital de Santo André |
|  | Comissão de Ética do Hospital Garcia de Orta  Pragal |
|  | Comissão de Ética do Hospital Distrital de Faro |
|  | Comissão de Ética do Centro Hospitalar de Cascais |
| **RUSSIAN Federation** | **IRB** |
|  | State Educadional Institution of Higer Professional  Education Russian State Medical University of  Ministry |
|  | CEC Petrovskiy blvd.8, 127051 Moscow.  Russian Cardiology Center |
|  | State Research Institute of Physicochemical Medicine of MoH of Russian Federation CCU |
|  | Educational Scientific Centre  of the Medical Centre of General Management  Department  of the President of the Russian Federation |
|  | CEC Petrovskiy blvd.8, 127051 Moscow.  Cardiology Research Institute named after Almazova. |
|  | CEC Petrovskiy blvd.8, 127051 Moscow. Ural State Medical Academy |
|  | CEC Petrovskiy blvd.8, 127051 Moscow. Tumen' Cardiology Centre |
|  | CEC Petrovskiy blvd.8, 127051 Moscow. F.I. Komarov |
| **SINGAPORE** | **IRB** |
|  | Clinical Trial Coordinating Centre  Health Sciences Authority  Singapore General Hospital Institutional Review Board |
|  | Clinical Trial Coordinating Centre  Health Sciences Authority  National Healthcare Group  Domain Specified Review Board C |
|  | Clinical Trial Coordinating Centre  Health Sciences Authority  Changi General Hospital  Institution Review Board |
| **SLOVAKIA** | **IRB** |
|  | Etická komisia  FNsP akademika L. Dérera |
|  | Etická komisia FN Nitra  Špitálska 6 |
|  | Etická komisia SÚSCH Pod Krásnou horkou 1 |
| **SLOVENIA** | **IRB** |
|  | Državna komisija za medicinsko etiko  Inštitut za klinično nevrofiziologijo  Klinični center Ljubljana |
| **SOUTH AFRICA** | **IRB** |
|  | Faculty of Health Sciences, University of Cape  Town |
|  | Research Ethics Committee, Faculty of Health  Sciences, Nelson R Mandela School of Medicine University of Kwazulu-Natal |
|  | Ethics Committee of the Faculty of Health Sciences, University of the Free State |
|  | The South African Medical Association Block F, Castle Walk Corporate Park |
|  | Human Research Ethics Committee: (Medical), University of the Witwatersrand, Johannesburg |
| **SOUTH KOREA** | **IRB** |
|  | St. Paul's Hospital IRB |
|  | Seoul National University Hospital IRB |
|  | Yeonsei Sevrance Hospital IRB |
|  | Kyungpook National University Hospital IRB |
|  | Chonnam National University Hospital IRB |
|  | Gachon Medical School Gil Medical Center IRB |
|  | IRB of Ewha Womans University Dongdaemoon  Hospital |
|  | IRB of Seoul National University Bundang Hospital |
|  | Korea University Guro Hospital IRB Young-Tae Bak |
|  | IRB of Inje University Busan Paik Hospital |
|  | Hallym University Sacred Heart Hospital IRB |
|  | Yeungnam University Medical Center IRB |
|  | IRB of Dong-A University Hospital |
|  | Wonju Christian Hospital, Yonsei  University Wonju College of Medicine IRB |
| **SPAIN** | **IRB** |
|  | CONSELLERIA DE SANITAT I CONSUM-SERVEI VALENCIA DE SALUT  Hospital “Dr. Peset”  COMITE ETICO DE INVESTIGACION CLINICA- VALENCIA |
|  | Comité Etico de Investigación Clínica  COMPLEJO HOSPITALARIO SAN CARLOS |
|  | Comitè ètic d´investigació clínica. ILLES BALEARS. Conselleria de Salut i Consum  Cecili Metel |
|  | Ethic Committee of Clinical Research  HOSPITAL VIRGEN DE LAS NIEVES DE GRANADA  Avda. Fuerzas Armadas |
|  | HOSPITAL CENTRAL DE ASTURIAS Comité Ético de Investigación Clínica Centro de Rehabilitación |
|  | HOSPITAL CLÍNIC I PROVINCIAL DE BARCELONA  Comité Ético de Investigación Clínica |
|  | HOSPITAL GENERAL UNIV. DE VALENCIAComité  Ético de Investigación Clínica  Fundación Hospital General Universitario de |
|  | HOSPITAL DE LEÓN  Comité Ético de Investigación Clínica |
|  | Hospital Virgen de la Victoria Campus Universitario Teatinos |
|  | HOSPITAL UNIVERSITARIO LA PAZ Comité Ético de Investigación Clínica  Escuela de Enfermería |
|  | COMITÉ ÉTICO DE INVESTIGACIÓN CLÍNICA División de Farmacia y Productos Sanitarios Servicio Gallego de Salud |
|  | HOSPITAL DE CRUCES  Comité Ético de Investigación Clínica  Pabellón de Administración, 5ª planta  Plaza de Cruces |
|  | HOSPITAL UNIVERSITARIO SAN JUAN Comité Ético de Investigación Clínica |
|  | CIUTAT SANITÀRIA I UNIVERSITÀRIA DE BELLVITGE  Comitè Ètic d’Investigació Clínica |
| **SWEDEN** | **IRB** |
|  | Regionala Etikprövningsnämnden i Uppsala, Medicincentrum, Akademiska sjukhuset |
| **SWITZERLAND** | **IRB** |
|  | Comitato etico cantonale c/o Sezione sanitaria |
|  | Ethikkommission des Kantons Thurgau  Postfach |
|  | Commission d'Ethique de la Recherche clinique de la  Faculté de Biologie et de Médecine  Décanat de la Faculté de Biologie et de Médecine |
|  | Ethikkommission beider Basel (EKBB) |
|  | Comitato etico cantonale c/o Sezione sanitaria |
| **TAIWAN** | **IRB** |
|  | Research Ethics Committee of National Taiwan  University Hospital |
|  | Institutional Review Board / Chang Gung Memorial  Hospital (IRB/CGMH) |
|  | Institutional Review Board / Chang Gung Memorial  Hospital (IRB/CGMH)  . |
|  | Institutional Review Board, Shin Kong Wu Ho-Su  Memorial Hospital |
|  | Mackay Memorial Hospital Institutional Review  Board |
| **THE NETHERLANDS** | **IRB** |
|  | Institutional Review Board  Academisch Medisch Centrum |
| **UKRAINE** | **IRB** |
|  | Legal address: 7, Grushevskogo str., Kiev, 01021, Ukraine; Ye.M.Gorban |
| **UNITED KINGDOM** | **IRB** |
|  | Northern & Yorkshire MREC  Sunderland Teaching Primary Care Trust (South  Office) Ryhope Hospital |
| **UNITED STATES** | **IRB** |
|  | Covent Health System IRB |
|  | Rapid City Regional Hospital IRB |
|  | St Joseph's Medical Center & St. Dominic's Hospital  Institutional Review Committee |
|  | Brotman Medical Center IRB |
|  | Virtua Health IRB and Our Lady of Lourdes IRB |
|  | Western Institutional Review Board |
|  | Brandon Regional Hospital IRB |
|  | Memorial Hospital of South Bend |
|  | Slidell Memorial Hospital IRB |
|  | Louisiana State University Health Science Center  Clinical Laboratories |
|  | University of Pennsylvania  Office of Regulatory Affairs |
|  | Amarillo IRB |
|  | Methedost Health Care System IRB |
|  | Western Institutional Review Board |
|  | Huntsville Hospital Institutional Review Committee |
|  | Mother Frances Hospital Regional Health Care  Center Institutional Review Board |
|  | Western Institutional Review Board |
|  | Institutional Review Board  Thomas Jefferson University |
|  | North Ridge Medical Center  Institutional Review Board |
|  | Univ. of Illinois College of Medicine at Peoria IRB |
|  | Human Studies Sub-Committee  Minneapolis VA Medical Center |
|  | Office of Human Research Protection |
|  | Bridgeport Hospital Institutional Review Board |
|  | Institutional Review Board  Lakeland Regional Medical Center |
|  | Western Institutional Review Board |
|  | Advocate Health Care  Institutional Review Board |
|  | Wuesthoff Hospital-Institutional Review Board |
|  | IRB for Human Subject Research for Baylor  C.O.M. |
|  | University of Cincinnati IRB |
|  | Western IRB |
|  | Crouse Hospital Institutional Review Board |
|  | Wilford Hall Medical Center IRB |
|  | VA Medical Center  Human Studies Comnmittee |
|  | Saint Mary's Regional Medical Center IRB |
|  | East Tennessee State University/VA IRB |
|  | St. Joseph Mercy - Oakland IRB/RC |
|  | Sentara Virginia Beach General Hospital IRB |
|  | Duke Institutional Review Board for Clinical |
|  | IRB, University of Chicago |
|  | Western Institutional Review Board |
|  | Medical Sciences Subcommittee for protection of human subjects research and graduate studies  Jackson Medical Towers |
|  | Institutional Review Board  Providence Saint Joseph Medical Center |
|  | Medical College Human Research Review  Committee |
|  | Insitutional Review Board  Corporate Medical Affairs |
|  | St. Vincent Hospital IRB |
|  | McGuire Institutional Review Board |
|  | Memorial Hospital at Gulfport IRB |
|  | University Hospital Institutional Review Board |
|  | St Vincent Hospital  Institutional Review Board |
|  | Midwest Regional Medical Center IRB |
|  | St. Joseph Mercy Hospital  Institutional Review Board |
|  | Luthern Health Network Institutional Review Board |
|  | Borgess Medical Center IRB |
|  | VA Pittsburgh Healthcare System IRB VA Pittsburgh Healthcare System |
|  | Allegheny General Hospital IRB |
|  | Biomedical Research Alliance of New York, LLC Institutional Review Board |
|  | Summa Health System Hospital IRB |
|  | Crozer-Chester Medical Center IRB |
|  | Institutional Review Board |
|  | INTEGRIS SW Medical Center IRB  . |
|  | The Christ Hospital IRB |
|  | Waukesha Memorial Hospital IRB |
|  | Aurora IRB |
|  | Combined Institutional Review Board |
|  | Research Committee  St. Joseph's Hospital Health Center |
|  | Danville Regional Medical Center  Institutional Review Board Committee |
|  | Des Moines Hospital Joint IRB |
|  | Wellmont Holston Valley Medical Center  Institutional Review Board |
|  | Hartford Hospital IRB Hartford Hospital |
|  | Health One Alliance Institutional Review Board |
|  | St. Luke's IRB |
|  | Health First Institutional Review Board |
|  | Abington Memorial Hospital IRB |
|  | Central Health Inc. IRB |
|  | Sacred Heart Hospital IRB |
|  | Florida Hospital |
|  | Cleveland Clinical Florida  Division of Research and Education |
|  | The George Washington University Medical Center  Office of Human Research |
|  | Institutional Review Board - The University of Texas  Health Center at Tyler |
|  | United Health Services Hospital IRB Wilson Memorial Regional Medical Center |
|  | University of Iowa IRB1 |
|  | Institutional Review Board  University of Massachusettes Medical School |
|  | Human Subjects Protection Program Office |
|  | St. Charles Medical Center  Institutional Review Board |
